# Advancing diagnostic certainty in Alzheimer’s disease: a synthesis of the diagnostic process

**DOI:** 10.3233/JAD-230186

**Published:** 2023-05-23

**Authors:** Jemma Hazan, Kathy Liu, Nick Fox, Robert Howard

**Affiliations:** 1 Division of Psychiatry, University College London, London; 2 Institute of Neurology, University College London, London, and Dementia Research Institute, UCL, London, UK

**Keywords:** Dementia, Alzheimer’s disease, Mild Cognitive Impairment, biomarkers, diagnosis, investigations

## Abstract

Changes in diagnostic certainty can be evaluated by assessing the impact of a diagnostic test in driving decision making. Diagnostic tests can be appraised using validated measures of accuracy, i.e.; sensitivity, specificity and positive or negative predictive values against a known reference standard. However, other less well formalised factors affect diagnostic certainty. These inputs are under-researched and more difficult to quantify. Clinicians assess the significance of available data in the context of their expertise, pre-diagnostic confidence, and background knowledge of populations and disease. Inherent qualities of the diagnostic test and an individual clinician’s interpretation of the meaning of test results will also affect the subsequent level of diagnostic certainty. These factors are only infrequently considered alongside the diagnostic accuracy of a test. In this paper, we present a model of the different processes which can affect diagnostic certainty in Alzheimer’s disease (AD). This model builds upon existing understanding and provides further insights into the complexity of diagnostic certainty in AD and how we might improve this.

## Introduction

Alzheimer’s disease (AD) is the most cause of dementia and comprises two-thirds of dementia diagnoses[1]. In the UK, most AD dementia diagnoses are made in memory services, which are based in the community within mental health services. The certainty of a secure AD dementia diagnosis is challenging and there is a 12-23% discrepancy between clinical and post-mortem confirmed AD diagnoses[2]. Clinicians in these services often have only limited access to specialist investigations such as amyloid-positron emission tomography (amyloid-PET) and cerebrospinal (CSF) fluid biomarkers[3,4]. The need for a secure and prompt diagnosis has never been more crucial in the context of the potential impact of disease modifying therapies (DMTs), which in current clinical trials have required AD pathology confirmation, via PET or CSF prior to commencement and will likely do so for clinical practice [5].

A clinician assesses the likelihood of a patient having a diagnosis of AD dementia by synthesising information gathered from several sources and assessments. There is an initial assessment where a history and cognitive examination are conducted. This may be followed by referral for diagnostic tests such as magnetic resonance imaging (MRI) or computed tomography (CT) of the brain, and a followup appointment at which test results are reviewed, and a final diagnosis is reached[6]. The diagnostic impact of laboratory or imaging investigations can be examined using well established tests of diagnostic accuracy and meta-analyses of published performance data[7]. However, there has been less research and emphasis placed on other contributors to a diagnosis of AD dementia, which we would argue are of at least equal importance.

**Fig.1:**
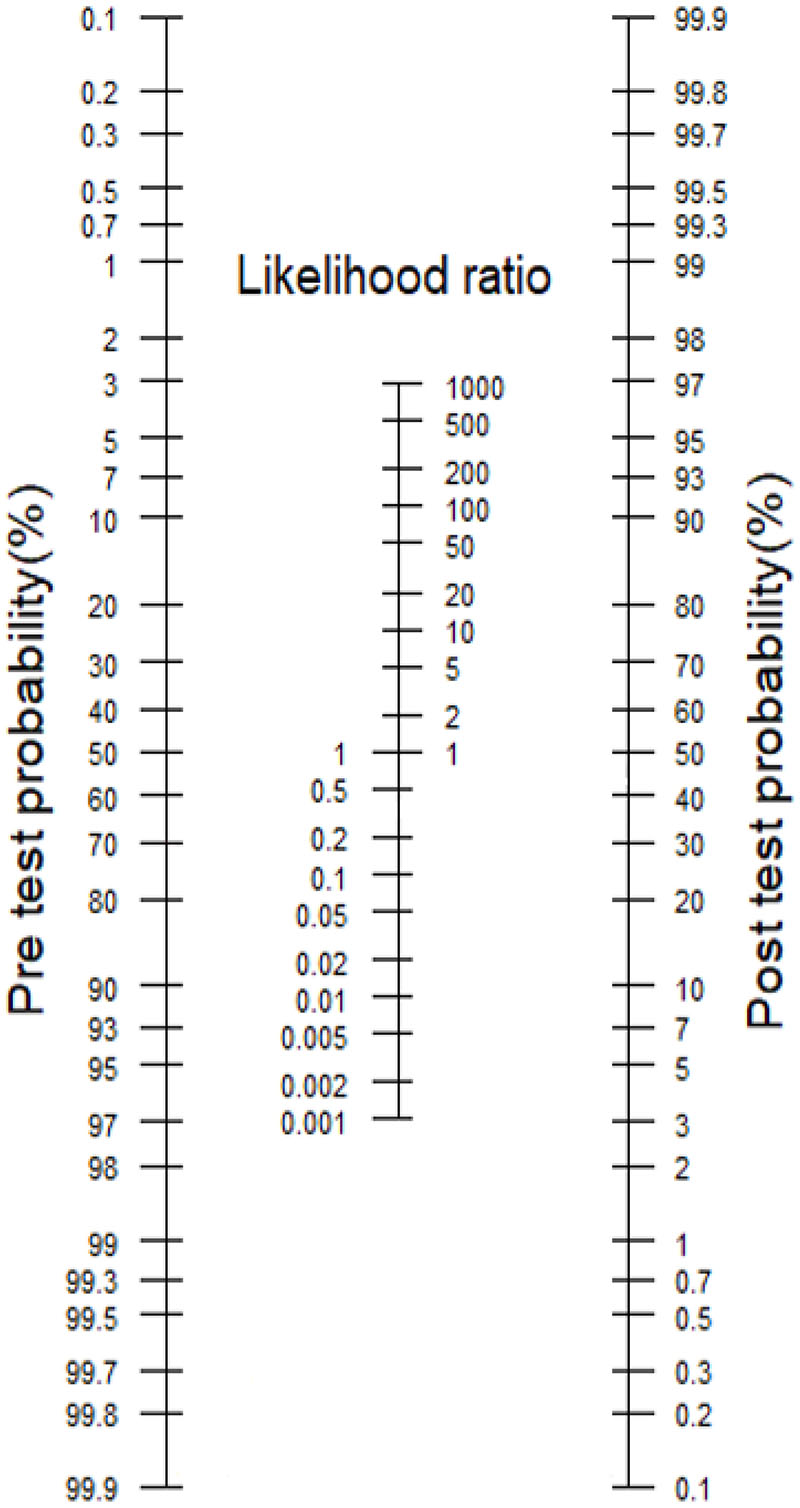
Fagan’s Nomogram[8] FAGAN’S NOMOGRAM

The probability of a patient having a condition of interest (e.g., AD), is quantified after the application of an investigation in evidence-based medicine[7]. This yields the post-test probability of a condition. Fagan’s nomogram was designed to integrate Bayes’ theorem into a graphical calculator (Fig.1). This provides a means for clinicians to quantify the post-test probability that an individual has a condition (such as AD), based on the pre-test probability (the probability of having the condition prior to the test), *and* their test result.

One can calculate the post-test probability of a condition such as AD by drawing a line from the pre-test probability to the likelihood ratio of a positive (LR+) or negative (LR-) diagnostic test and extending the line to intersect with the post-test probability axis, which gives the post-test probability.All the elements which contribute to Fagan’s nomogram are underpinned by the diagnostic accuracy of the test, as measured by LR+ or LR- (i.e., whether the test had a binary outcome).

However, Fagan’s nomogram is not able to fully account for other influences on the diagnostic certainty of AD. These can be clustered into the elements of the nomogram: Pre-test probability, Likelihood Ratio and Post-test probability.

## Pre-Test Probability

### Prevalence

The pre-test probability is defined as the probability that the person has AD and is influenced by the prevalence of the disease in the population of interest. For example, the prevalence rate of dementia among older people in the UK is estimated to be 7.1%[11]. This would be the pre-test probability for a screening test applied to older adults in the UK. The age-specific prevalence of AD almost doubles every 5 years after the age of 65. It is estimated that 10% of people over the age of 65 are symptomatic AD cases and this rises to >1/3 of those aged >85 years[12,13]. Therefore, the pre-test probability of AD is strongly influenced by the age of the patient assessed. For a patient under evaluation in the memory clinic, the clinician may assign an AD probability that is different from the probability assigned by the primary care physician referring the patient. Therefore, for an individual who attends the memory clinic for assessment and is considered for a test, the pre-test probability of AD in symptomatic (cognitively impaired) older individuals is likely to be even higher. The pre-test probability will vary between clinics and over time. For example, with greater public awareness and anxiety around symptoms and diagnosis there may be more referrals of those who do not have the disease in question.

### Clinician Confidence

The clinician’s degree of belief in the diagnosis of AD represents the first-order probability. One way of expressing confidence in a Bayesian framework is through second-order probabilities, these represent the clinician’s degree of belief in the first-order probability[14]. This provides a means of incorporating a type of judgmental behaviour, i.e., for the clinician this is an evaluation of the adequacy of their own knowledge. Such judgments by the clinician will have ramifications for whether to seek further information, length of information analysis, and when to reach a diagnostic decision. The clinician’s degree of confidence and strength of suspicion in the diagnosis will impact on the first-order probability of an AD diagnosis.

Clinician-related factors are an important consideration and are influenced by clinical experience and a clinician’s personal impression after history and examination[15]. The confidence of the clinician may be influenced by their seniority and training, with a positive correlation between years of experience and degree of confidence[16]. Some individual clinicians are more confident in making diagnoses and tolerating diagnostic uncertainty. Clinician-related certainty may impact on both the decision to refer for diagnostic testing and the quantity or type of investigations requested[17]. These decision-making processes have been less robustly studied.

Diagnostic calibration describes the relationship between a clinician’s confidence in the accuracy of their diagnosis and their actual accuracy[18]. If a clinician is overconfident, too much weight may be placed on their unconscious biases and this may lead to diagnostic error[19]. Clinician under confidence may lead to delayed diagnosis or overzealous ordering of additional investigations. In one study using simulated case vignettes, diagnostic calibration had greater discordance when the difficulty of the case scenario increased[20]. This was due to a decrease in the accuracy of diagnosis for these more difficult scenarios, while clinician confidence remained static.

### Access to Investigations

In UK memory services, there is limited access to more specialised investigations such as amyloid-PET or CSF biomarkers‥ Only 2% of patients are referred to neurology clinics for additional specialist assessment[3]. A clinician’s experience with, not just access to, a test may increase awareness of its strengths and weaknesses and affect the decision to use the test. As a result, clinicians’ familiarity in the use of amyloid-PET/CSF and subsequent interpretation of results is lower than for diagnostic tests, such as CT/MRI neuroimaging, which has greater accessibility[21]. Several blood-based AD biomarkers are now available in the research setting and correlate well with their CSF counterparts[22]. A blood test that was cheap and simple to carry out would increase the accessibility of AD biomarker testing[23].

### Typical vs A Typical AD Presentations

The most common clinical presentation of AD is one of a slowly progressive amnestic syndrome. However, a proportion of patients present with atypical phenotypes. These include dysfunction in visual, language, executive, behavioural, or motor domains. Early-onset AD is defined as symptoms onset before the age of 65 years[24]. Atypical presentations are more common in early-onset AD. Much of clinical diagnosis within medicine is based on pattern recognition[25]. The combination of a younger onset, in itself rare and an atypical presentation may lead to a reduction in such cases of AD being identified and a subsequent delay in diagnosis[26]. In a cohort of young-onset cases with neuropathological AD confirmation, there was a misdiagnosis rate of 53% of those with atypical presentations versus 4% of patients with a more typical presentation[27,28].

#### Clinical Assessment

The quality of the clinical assessment and access to a comprehensive history will impact on the pre-test probability of a diagnosis of AD. This can prove challenging when patients attend clinic on their own or may not have an available or reliable informant. Access to a collateral history is essential to making a diagnosis of AD. Patients with cognitive impairment are less likely to be able to convey information regarding their premorbid level of function and cognition and how this has changed over time[29]. In addition, the patient may lack insight into the degree of difficulties they are experiencing. Despite collateral history being a cornerstone of diagnostic assessment, there is little research in the field or formal guidance on how to take a thorough collateral informant history[30,31]. There are some scales, such as the Clinical Dementia Rating (CDR) which have guidelines and training[32]. In a UK audit of dementia care, out of those classified as having dementia, only one-third had an informant history recorded[33].

In one study which used simulated case vignettes, clinicians were presented with a range of clinical scenarios along with CSF AD biomarkers results[34]. If the clinicians were shown an AD clinical presentation and consistent AD CSF results, this resulted in a significantly increased odds of an AD diagnosis. However, if clinicians were provided with borderline CSF values, they instead relied on other clinical information to reach a final diagnosis. When clinicians viewed a mild AD clinical presentation with normal CSF results, the diagnosis they picked was one of unknown aetiology. Finally, when clinicians were given an ambiguous clinical presentation with AD CSF biomarker results, there was an increased likelihood to give an AD diagnosis. These cases highlight both the impact of investigation results on final diagnosis, but more importantly the need to interpret the investigation result in the context of the overall clinical picture.

## Likelihood Ratio

### Diagnostic Accuracy

The likelihood ratio (LR) is defined in group-based statistics as the ratio of an expected test result in those with a disease to those without the disease[9]. It is calculated from the diagnostic accuracy of the test, i.e., its sensitivity (how well it can identify true positives) and specificity (how well it can identify true negatives). However, when applying Bayes' theorem in clinical practice, it is crucial to consider individual likelihoods, which refer to the conditional probabilities of obtaining a positive or negative test result given the presence or absence of the disease, respectively. This distinction is significant as group-based test accuracy statistics, such as the likelihood ratio, are not fixed, but rather known to vary depending on various factors such as the testing setting, prior tests, and patient characteristics like sex and age[35]. In other words, the LR is the ability of a test to discriminate between having an AD diagnosis or not.

The LR will vary for a positive test result (LR+) or a negative test result (LR-). LR+ informs the clinician about how much more likely the positive test result is to occur in people with AD compared to those without AD. The higher the LR+, the more likely AD is present. A LR+ > 10 is considered to reflect a good diagnostic test and that a positive test result has a significant contribution to the diagnosis and increases the post-test probability. A LR- provides a means of ruling out a diagnosis of AD. A LR- < 0.1 reflects a good diagnostic test. The lower the LR-, the larger the impact of the test to rule out and reduce the post-test probability of the subject having AD.

An uncertain pre-test probability is the best case for use of a diagnostic test with sufficiently high LR + or low LR-. A very high or low pre-test probability will not be appreciably affected irrespective of the likelihood ratio of the test[35,36]. This is particularly important when considering the use of costly or invasive investigations such as amyloid-PET[37].

### Diagnostic Test Qualities

There are inherent differences in the properties of diagnostic tests used in the assessment of AD. CT and MRI neuroimaging provide a means of ruling out reversible causes of dementia and assist in pathological subtyping[6]. This includes assessing the volume of the medial temporal lobe, which is a biomarker for AD. The Scheltens visual rating scale for degree of medial temporal lobe atrophy (MTA) is used in clinical practice and a MTA cut-off value of ≥2 (0-4) in the age group 75–84years provides a sensitivity of 73.7%, specificity of 76.2%, LR+ 3.10 and LR- 0.35 for distinguishing between AD and those who are cognitively normal[38].

**Fig.2:**
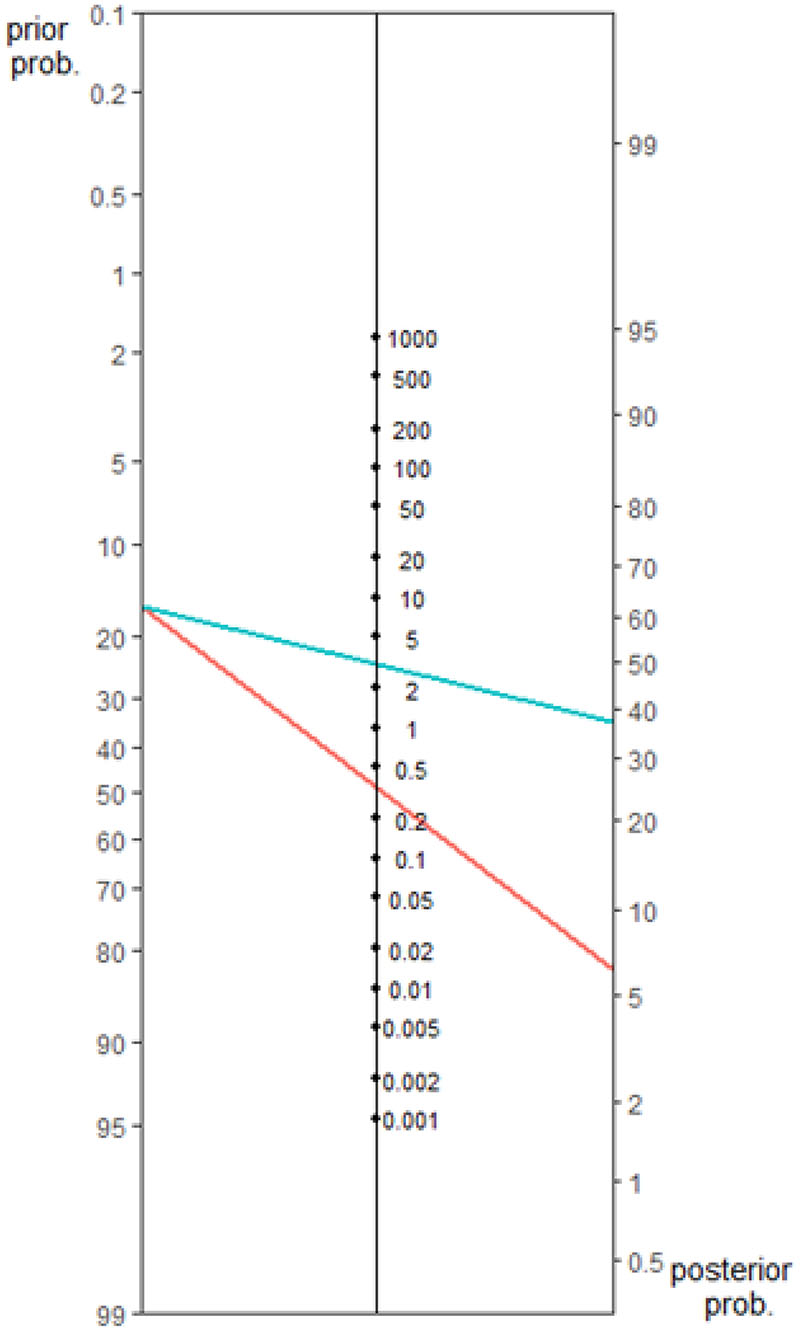
Nomogram of medial temporal lobe atrophy (MTA) score in MRI neuroimaging in the diagnosis of Alzheimer’s disease using a cut score of ≥2 derived from a validated visual rating system, in a 0 to 4 scale, for rating atrophy of medial temporal lobe structures[39].

An example nomogram for the use of medial temporal lobe atrophy (MTA) score in MRI neuroimaging in the diagnosis of Alzheimer’s disease highlights the impact of LR+ (blue line) or LR-(red line) on post-test probability (Fig 2).

The pre-test probability in this example is based on age-based prevalence statistics. If the population of interest were between the ages of 75–84 years, the prevalence of AD would be 15–18% [40]. A MTA score **≥**2 is defined as a positive result and would have a sensitivity of 73.7% and a specificity of 76.2%[39]. In this nomogram, the pre-test probability is set at 16%. With a positive result, calculated using an MTA cut-off value of ≥2, the positive likelihood ratio (LR+) is 3.10 CI (2.66-3.61), and the post-test probability is 37% CI (34-41) (blue line). If the result was negative, calculated using an MTA cut-off value <2, the negative likelihood ratio (LR-) is 0.35 CI (0.27-0.45) and the post-test probability is 6% CI (5-8) (red line).

However, in memory services clinicians do not usually have access to the CT or MRI images and are reliant on the quality of the radiology report. The report may not include a formalised rating scale of atrophy. While MRI provides a more anatomically detailed image than CT, it is more expensive and requires patients to tolerate lying still in a noisy scanner for >30 minutes. Less than 20% of patient reviewed in memory services have a MRI scan[41].

Appropriate use criteria have been published to guide clinicians on when to consider specialist investigations such as amyloid-PET or CSF[37,42]. Examples of appropriate use criteria for CSF testing include meeting the core clinical criteria for a probable AD dementia with typical age of onset or having symptoms suggesting a possible AD dementia[42].

The National Institute for Health and Care Excellence (NICE), which publishes guidelines on the use of health technologies within the National Health Services (NHS) in England and Wales, suggest considering their use when the diagnosis of dementia is uncertain[6]. Amyloid-PET is costly and sometimes requires a patient to remain still for a long period of time while undergoing intravenous infusion of a radioactive isotope[43]. Lumbar puncture for CSF biomarkers is invasive and a clinician will need to consider if this is clinically appropriate in patients who may not be able to tolerate the procedure or have contra-indications e.g. have bleeding disorders or are taking anticoagulant medicines[42,44].

Amyloid-PET can provide a dichotomous test result reported as either positive or negative[45]. This increases the ease of interpretation. However, the result is only indicative of the degree of brain amyloid burden, which can occur in cognitively healthy older adults. Amyloid burden rises with age and there is a long pre-clinical period of amyloid positivity. While an amyloid positive scan can identify amyloid beta pathology in the brain it provides little precision in predicting when someone would be symptomatic. In a prospective cohort study the average interval between amyloid positivity, defined as a standardized uptake value ratio (SUVR) threshold of 1.2, and displaying symptoms of AD was ~12 years[46]. An individual can be positive on PET in non-AD such as dementia with Lewy bodies[47]. Other investigation results are less clear-cut. While there are established cut-points for CSF biomarker results, the biomarker concentrations are continuous variables and results may be ambiguous and indeterminate, sitting between a suggestive ‘AD-like’ result and a ‘non-AD like’ result[48].

Further validation studies are needed to elucidate how demographic factors such as ethnicity and age may affect biomarker concentrations and limit the applicability of a biomarker result in different populations[49]. It is also important to consider how cut-off values are determined as this will affect the sensitivity, specificity and likelihood ratio of a diagnostic test[50].

## Post-Test Probability

The post-test probability is the likelihood of a person having a diagnosis of AD based on their pre-test probability and test result.

## Clinical Utility

In a meta-analysis of the clinical utility of CSF biomarkers in the assessment of patients under evaluation for AD, CSF biomarkers improved clinicians’ diagnostic confidence with a pooled mean increase of 14%, pooled percentage change in diagnosis of 25% and a pooled proportion of patients whose management was subsequently changed of 31% [51]. The change in clinicians’ confidence after receiving CSF results was inversely proportional to the initial pre-test confidence level. The lower the clinicians’ pre-test confidence, the greater the percentage change in confidence once they received the biomarker result. The impact of amyloid-PET on clinicians’ change in confidence level ranged between 16 to 44%[52].

## Test Interpretation

The clinician is required to integrate their assessment and the investigation tests results to formulate a diagnosis. There are a range of scenarios where the clinician will need to manage uncertainty or conflict, such as when the result of a test or tests does not meaningfully increase (or reduces) diagnostic certainty, or if multiple test results, e.g. MRI and CSF / imaging and fluid biomarkers conflict with each other.

While the National Institute on Aging and Alzheimer Association (NIA-AA) criteria states that biomarkers facilitate the accurate and timely diagnosis of AD, they do not provide information on how clinicians should handle conflicting or indeterminate biomarker results[53]. There is variation in clinicians’ approach to biomarker interpretation. There may be differences in clinicians’ capacity to manage conflict between clinical impression and biomarker profile when interpreting investigation results. This may be related to clinical factors such differences in reasoning and decision-making, experience, and training in handling conflict.

## Case Scenarios

The following case scenarios aim to demonstrate how factors in this model interact. Baseline pre-test probabilities may differ based on the clinical presentation/assessment. The diagnostic test result, which may be positive, negative, or intermediate/conflicting can impact post-test probabilities. There is additionally a cumulative impact of consecutive investigations and their interpretation by the requesting clinician. Clinicians do not tend to quantify their pre-test probability in any formal way and the chosen pre-test probabilities in these scenarios represent ‘low’ ‘uncertain’ or ‘high’ pre-test diagnostic confidence.

1. A 70-year-old man presents with a 1-year history of gradual cognitive decline with episodic memory problems. The GP referral describes him having trouble in recalling recent events including conversations and news items on the television, however the patient does not feel he has much of a problem. His clinician is unable to garner collateral from an informant. His Mini Mental State Examination (MMSE) score is 27/30. After initial assessment and prior to ordering of any investigations, the clinician concludes that the probability of the patient having AD is low (pre-test probability estimated at 20%).

**Fig.3:**
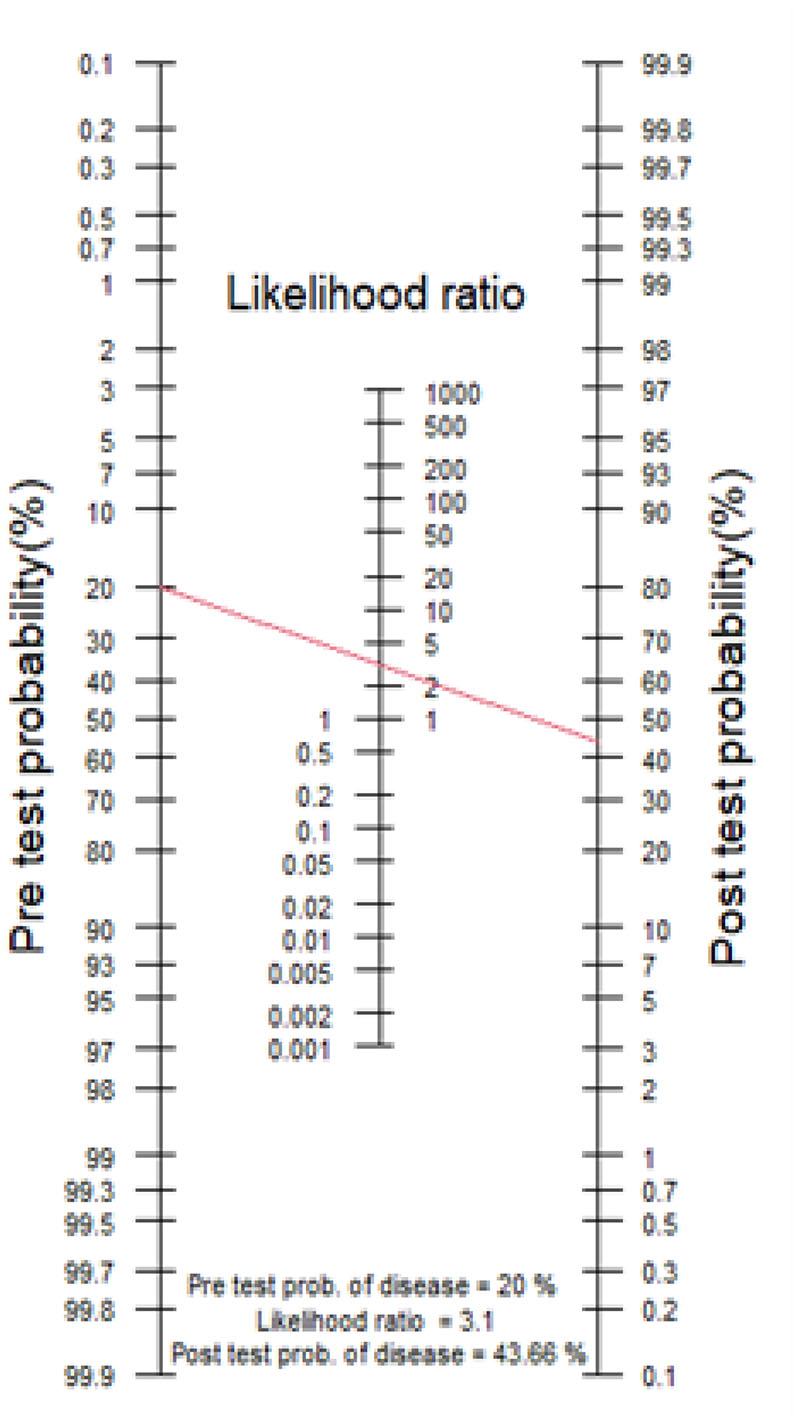
Case Scenario 1 Nomogram. Pre-test probability of 20% and LR+ of 3.1 and a post-test probability of 44% (red line).

The clinician decides to invite the patient to have an MRI brain, which shows a ‘positive’ result, i.e. the MTA score is 3. With a positive likelihood ratio (LR+) value of 3.10 (calculated using an MTA cut-off value of ≥2 with sensitivity 73.7% and specificity 76.2%[39]**),** the corresponding post-test probability increases to 44% (Fig 3.) This represents clinical diagnostic uncertainty, and the clinician may then decide to order further investigations if available. In contrast, had the test been negative, the clinician’s initial impression would have been confirmed as the post-test probability would have decreased to 8%.

2. A 74-year-old man presents with a 2-year history of gradual cognitive decline with episodic memory problems. He had been having trouble in recalling recent events including conversations and news items on the television. His wife is present and provided collateral, revealing that he had gradually become more repetitive in conversation. He was struggling to use the computer to surf the internet and check his email. His MMSE score is 25/30. After initial assessment and prior to ordering of any investigations the clinician concludes that the probability of the patient having AD is high (estimated pre-test probability 70%).

The clinician decides to invite the patient to have an MRI brain, which is ‘negative’, i.e. the MTA score is 1. The negative likelihood ratio (LR-) of the test is 0.35 (calculated using using an MTA cut-off value of ≥2 with sensitivity 73.7% and specificity 76.2%[39]**),** and the post-test probability decreases to 45%. This represents clinical diagnostic uncertainty, and the clinician decides to refer him for further investigation. For comparison, had the test been positive, the clinician’s initial impression would have been confirmed as the post-test probability would have increased to 88%.

3. The same 74-year-old man from case scenario 2 above is referred for an amyloid-PET scan, which shows a ‘positive’ result. The calculated positive likelihood ratio (LR+) of the PET scan is 6 (calculated from values for sensitivity 90% and specificity 85%[54]), which increases the post-test probability to 85%, i.e. the probability of having AD is considered to be high. This gentleman receives a diagnosis of AD dementia. In contrast, had the test been negative, the post-test probability would have decreased to 9%, which would have influenced subsequent clinical decisions.

## Discussion

The diagnosis of AD provides many challenges for clinicians. While Fagan’s nomogram can help to interpret the utility of a diagnostic test, there are other factors which influence the diagnostic certainty of AD. These have been incorporated into this model. Further work is required to elucidate the impact of clinician related factors on this process.

The advent of novel AD biomarkers and excitement around the prospect of access to blood-based biomarkers in UK memory services and potential AD course-modifying treatments has heralded a new era in dementia diagnosis. With this progress comes the need to ensure that diagnostic tests are used appropriately, and results are interpretated as part of the entire clinical picture. Blood-based biomarkers could provide clinicians with widely available access to a panel of new diagnostic tests, collected from a single plasma sample[5]. However, even if one new blood biomarker was introduced, clinicians will be required to integrate a greater degree of information in their assessment. Currently a clinician implicitly combines and integrates or discards multiple pieces of information. These include patient demographics e.g., age; patient and collateral histories; and the examination of the patient, which may include bedside tests and investigations. Only now there will be a greater dimensionality to these investigations. Not only can we assess markers of disease *effect*, such as the degree of white matter hyperintensities or atrophy on a brain scan, but we would have access to markers of molecular pathology *cause*, which may or may not be having an effect and may or may not align with other results or the clinician’s clinical impression. This should have implications for further research on biomarker interpretation, managing conflicting results and may lead to the introduction of decision support tools.

While much consideration is given to the diagnostic accuracy of an investigation, several other core elements affect the diagnostic certainty of an AD diagnosis. We suggest that appropriate training packages and guidelines for clinicians are developed that incorporate these concepts. The use of e-tools would be valuable in training clinicians to understand and interpret blood-biomarkers using a range of simulated case vignettes. It is also important for clinicians to incorporate longitudinal followup in their assessment when there is diagnostic uncertainty. Any diagnosis should be re-considered if the clinical disease progression and or treatment response is not consistent with the anticipated course.

In conclusion, while a definitive diagnosis of AD can only be made with certainty on post-mortem neuropathologic evaluation, managing uncertainty and the appropriate use of diagnostic tests based on the clinical presentation is a core skill for clinicians seeking to achieve a diagnosis in life. Clinicians will require an improved understanding of pre-test and post-test probabilities, the appropriate use of novel diagnostic tests and how to manage uncertainty. These skills will become increasingly important against a back-drop of an increased need for diagnostic certainty with the advent of pathology-specific DMTs.

## Figures and Tables

**Fig.4:**
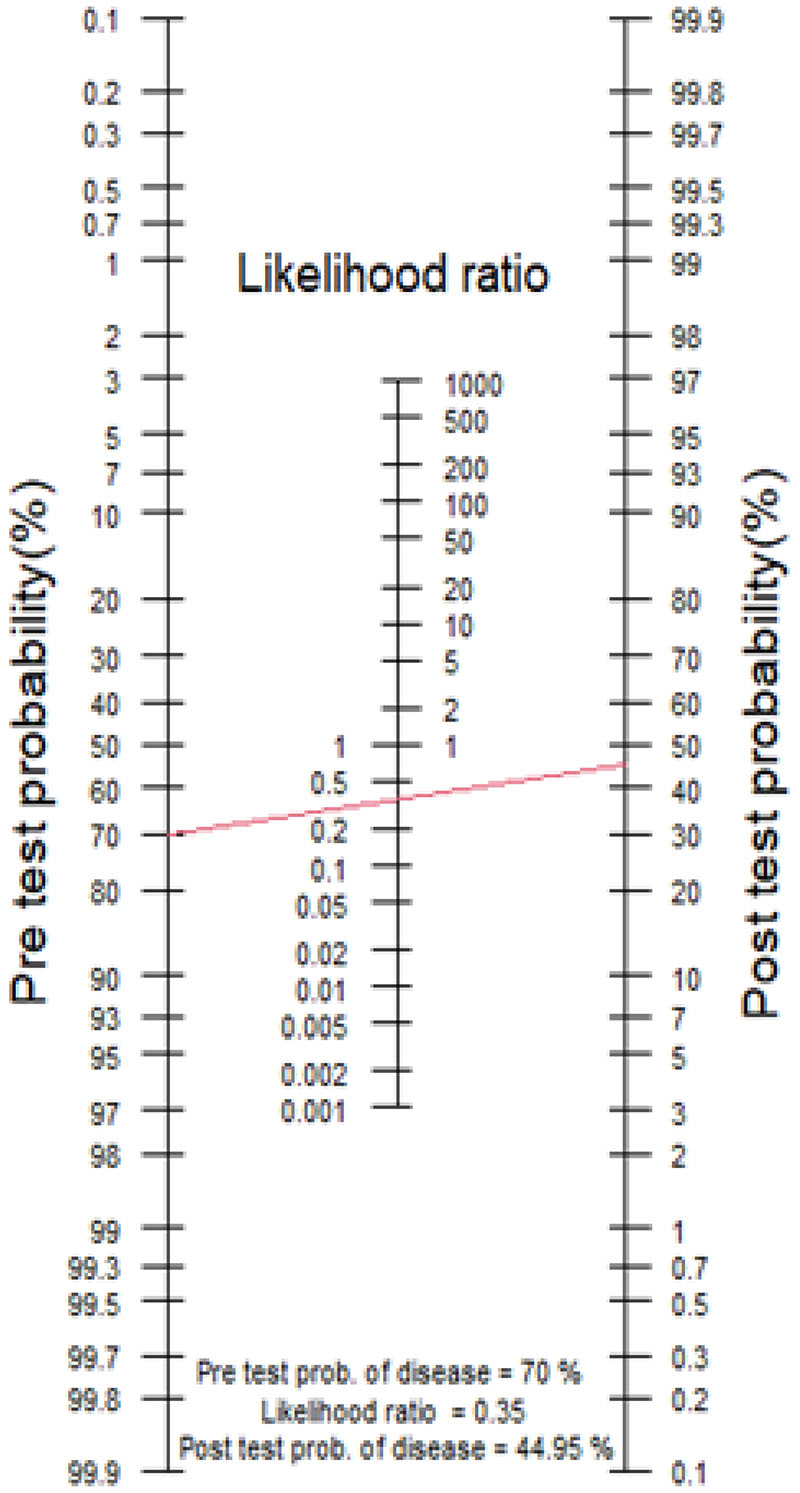
Case Scenario 2 Nomogram. Pre-test probability of 70% and LR- of 0.35 and post-test probability of 45% (red line).

**Fig.5:**
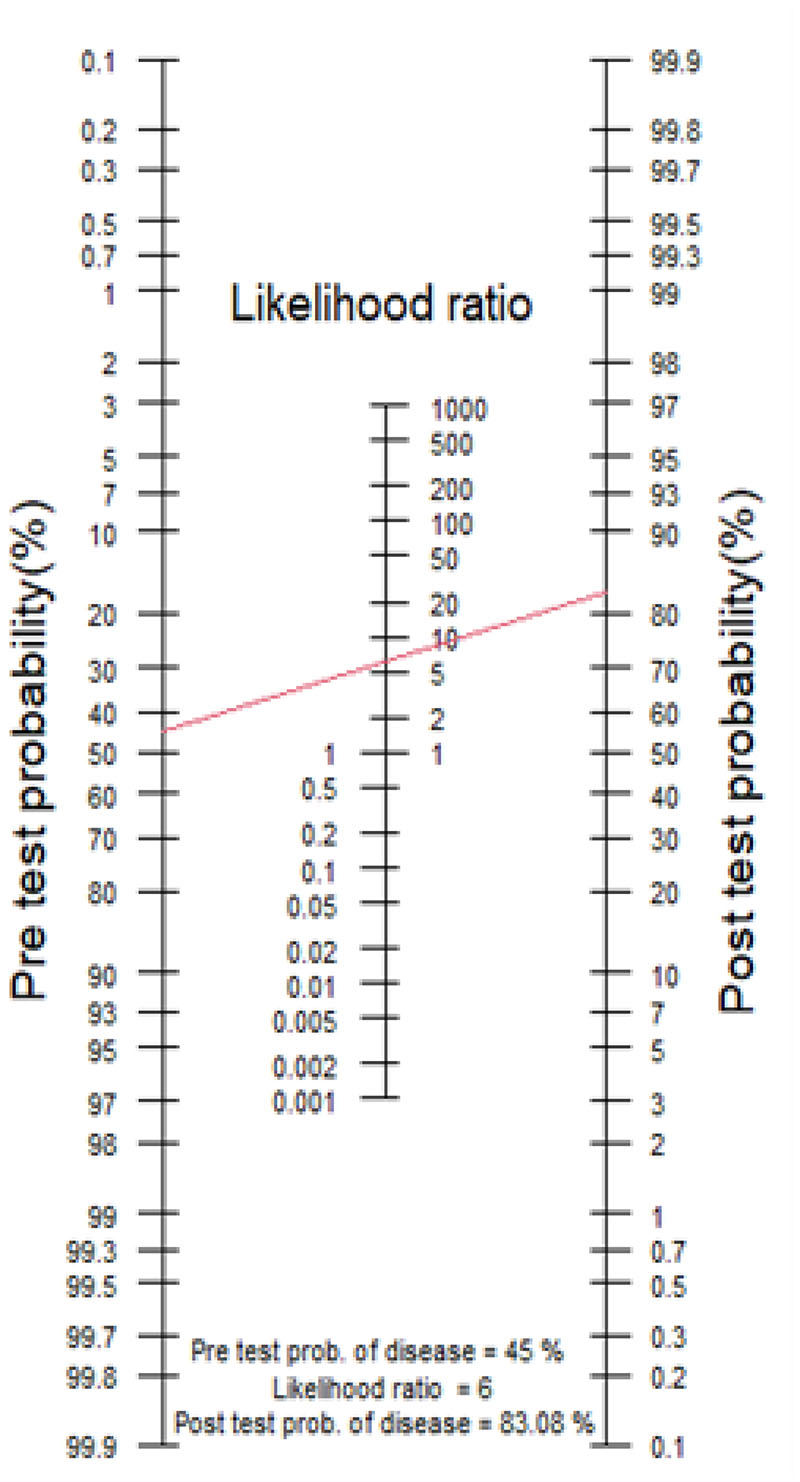
Case Scenario 3 Nomogram. Pre-test probability of 45% and LR+ of 6 and a post-test probability of 83% (red line).

**Table 1:** Summary of key terms

Term	Description
**Sensitivity**	The proportion of patients with a positive test result in a group of patients with the disease, i.e. ‘true positives’[9]
**Specificity**	The proportion of patients with a negative test result in a group of patients without the disease, i.e. ‘true negatives’[9]
**Pre-test Probability**	The probability of the person having the disease before the test
**Likelihood Ratio**	The ratio of an expected test result in those with a disease to those without the disease
**Positive Likelihood Ratio (LR+)**	The probability that a person with the disease has a positive test (true positive, or sensitivity) divided by the probability that a person without the disease has a positive test (false positive, or 1 - specificity).
**Negative Likelihood Ratio (LR-)**	The probability that a person with the disease has a negative test (false negative, or 1 - sensitivity) divided by the probability that a person without the disease has a negative test (true negative, or specificity)[10]
**Post-test Probability**	The probability of the patient having the disease after the test
